# Evidence of Bioactive Compounds from *Vernonia polyanthes* Leaves with Topical Anti-Inflammatory Potential

**DOI:** 10.3390/ijms17121929

**Published:** 2016-12-01

**Authors:** Kamilla C. M. Rodrigues, Lucas A. Chibli, Bruna C. S. Santos, Vanessa S. Temponi, Nícolas C. C. Pinto, Elita Scio, Glauciemar Del-Vechio-Vieira, Maria S. Alves, Orlando V. Sousa

**Affiliations:** 1 Department of Pharmaceutical Sciences, Faculty of Pharmacy, Federal University of Juiz de Fora, Rua José Lourenço Kelmer, s/n, Campus Universitário, Juiz de Fora, Minas Gerais 36036-900, Brazil; kamillacoelho@yahoo.com.br (K.C.M.R.); lucano.farm@gmail.com (L.A.C.); brunaceleida@gmail.com (B.C.S.S.); vanessatemponi@hotmail.com (V.S.T.); glauciemar@gmail.com (G.D.-V.-V.); alves_ms2005@yahoo.com.br (M.S.A.); 2 Department of Biochemistry, Institute of Biological Sciences, Federal University of Juiz de Fora, Rua José Lourenço Kelmer, s/n, Campus Universitário, Juiz de Fora, Minas Gerais 36036-900, Brazil; nickbioquimica@hotmail.com (N.C.C.P.); elita.scio@ufjf.edu.br (E.S.)

**Keywords:** *Vernonia polyanthes*, asteraceae, inflammation, anti-inflammatory agents, myeloperoxidase, *N*-acetyl-β-d-glucosaminidase, ear edema

## Abstract

*Vernonia polyanthes* Less. (Asteraceae), popularly known as “assa-peixe”, is a plant species used in Brazilian traditional medicine for the treatment of cutaneous damage, cicatrization, inflammation, and rheumatism. Based on these ethnopharmacological findings, the current study evaluated the topical anti-inflammatory effects of the hexane (HEVP) and ethyl acetate (EAEVP) extracts from *V. polyanthes* leaves in experimental models of skin inflammation. Chemical characterization was carried out by HPLC–UV/DAD analysis. Anti-inflammatory activity was evaluated using Croton oil-, arachidonic acid (AA)-, phenol-, ethyl phenylpropiolate (EPP)-, and capsaicin-induced ear edema models in mice. Histopathological evaluation and measurements of myeloperoxidase (MPO) and *N*-acetyl-β-d-glucosaminidase (NAG) enzymes were also performed. Rutin, luteolin, and apigenin were identified in EAEVP. Topically applied HEVP and EAEVP significantly (*p* < 0.05, *p* < 0.01 or *p* < 0.001) reduced edema induced by five different irritants at the doses tested (0.1, 0.5 and 1.0 mg/ear). Histopathological analysis revealed a reduction of edema, inflammatory cell infiltration, and vasodilation. In addition, the enzymes activity (MPO and NAG) in the ear tissues was reduced by the topical treatment of HEVP and EAEVP (*p* < 0.05, *p* < 0.01 or *p* < 0.001). The results suggest that *V. polyanthes* leaves are effective against cutaneous damage, which support its traditional use and open up new possibilities for the treatment of skin disorders.

## 1. Introduction

The skin is the largest organ in the human body, and a highly metabolic tissue that serves as the protective layer for internal organs, since it provides the first line of defense against injury and aggressive environmental agents [1,2]. These agents, such as microorganisms, parasites, particles, irritants, and pollutants, are potent activators of inflammation that disturb homeostasis and promote several dermatological pathologies [3,4]. Skin manifestations can also be caused by systemic disorders, use of medications, and several other factors [5,6,7]. As a consequence, the skin diseases are responsible for numerous visits to health care services that are associated with hospitalization, morbidity, and mortality, compromising the physical and emotional well-being and the quality of life of the individual. In addition, since the 70s, epidemiological studies have shown an increasing number of human skin disease cases [8,9,10]. In Brazil, for example, 57,343 cases of skin diseases had been identified, with acne being the most frequently disorder observed [11]. Sexually transmitted diseases, allergic skin diseases, unspecified dermatoses, leprosy, seborrhea, and related diseases were also identified [12]. Based on these reports, it is evident the importance of the search for new and alternative therapeutic options for the care of different skin conditions.

Skin inflammatory disorders as psoriasis and allergic dermatitis are treated with appropriate medicines [1,13]. However, the different presentations of skin diseases involve a variable approach to treatment based on the severity of the case. According to a significant literature, topical agents have been used for the treatment of mild problems and as adjuvant in moderate-to-severe conditions, since they can reduce the amount of phototherapy or systemic drugs [14,15]. In the case of psoriasis, for instance, although there is a wide range of available therapies, the use of topical products has facilitated the maintenance of treatment [14]. Considering this evidence, natural products can represent an important source for the development of new drugs for the treatment of skin disorders related to inflammatory conditions [16].

Among the many plants found in Brazilian biodiversity, *Vernonia polyanthes* Less, belonging to the Asteraceae family, has been traditionally used as a diuretic, hypotensive, antiulcerogenic, antirheumatic, antimicrobial, cicatrizing, cutaneous damage, anti-inflammatory [17], and for the treatment of malaria and fever [18]. Focusing on the pharmacological investigations, this species has shown antiulcer [19], antinociceptive, anti-inflammatory [17], antihypertensive [20], antibacterial [21] and antifungal [22] activities. These properties can be related to the presence of compounds such as 3,7-dimethoxy-5,3′,4′-trihydroxyflavone, 3′,4′-dimethoxyluteolin, 3,5-di-*O*-(*E*)-caffeoylquinic acid, 4,5-di-*O*-(*E*)-caffeoylquinic acid, luteolin, quercetin, protocatechuic acid, quercetin-3-*O*-β-glucoside, apigenin, isorhamnetin, chrysoeriol-7-*O*-glycuronyl, and acacetin-7-*O*-glycuronyl; as well as sesquiterpenes lactones glaucolide A, piptocarphin A, and piptocarphin B, identified in *V. polyanthes* leaves [18,23]. In addition, phytochemical screening of the ethanol extract from leaves showed the presence of flavonoids, tannins, coumarins, terpenoids, sterols, saponins, and alkaloids [17]. Natural compounds, such as flavonoids and terpenes, can act by different mechanisms of action, inhibiting the synthesis or expression of inflammatory mediators [24,25,26].

Considering the medicinal applications, especially for the treatment of rheumatism, cicatrization, cutaneous damage and inflammation, as well as the pharmacological evidences, the present investigation was designed to evaluate the topical anti-inflammatory effect of the hexane (HEVP) and ethyl acetate (EAEVP) extracts of *V. polyanthes* leaves using five models of inflammation in the mouse ear. In addition, chemical characterization of HEVP and EAEVP was conducted in order to identify possible bioactive compounds in these extracts.

## 2. Results

### 2.1. Chemical Characterization by HPLC

**Figure 1:**
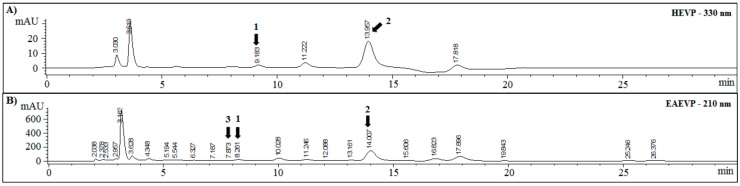
Chromatograms of HEVP and EAEVP. (A) HEVP-330 nm; and (B) EAEVP-210 nm. (**1**) luteolin (2-(3,4-dihydroxyphenyl)-5,7-dihydroxy-4-chromen-one); (**2**) apigenin (5,7-dihydroxy-2-(4-hydroxyphenyl)-4*H*-1-benzopyran-4-one); and (**3**) rutin (2-(3,4-dihydroxyphenyl)-5,7-dihydroxy-3-[α-l-rhamnopyranosyl-(1→6)-β-d-glucopyranosyloxy]-4*H*-chromen-one).

**Figure 2:**
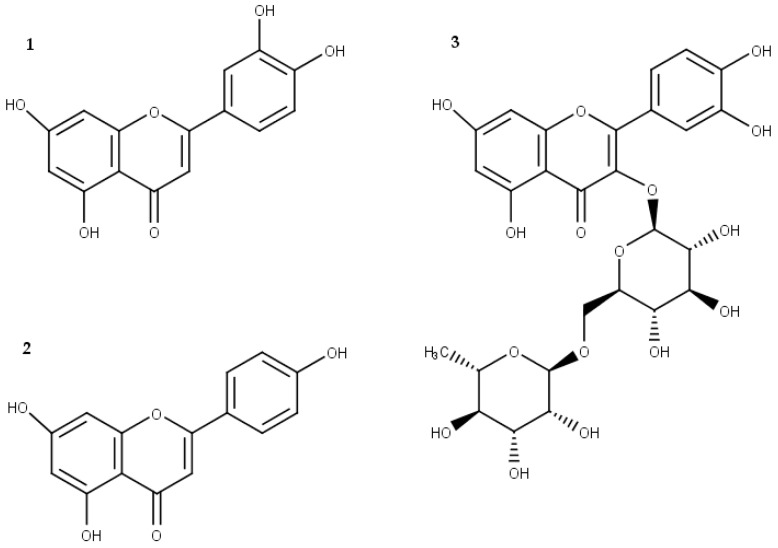
Chemical structure of the main flavonoids found in EAEVP. (**1**) Luteolin; (**2**) apigenin; and (**3**) rutin.

HPLC fingerprintings of HEVP and EAEVP at 210 and 330 nm, supported by t_R_ and UV spectrum data from standard substances, are showed in Figure 1. HEVP revealed spectra with characteristic of flavonoids, which luteolin or 2-(3,4-dihydroxyphenyl)-5,7-dihydroxy-4-chromen-one (**1**) (peak 1, t_R_ = 9.1 min), and apigenin or 5,7-dihydroxy-2-(4-hydroxyphenyl)-4*H*-1-benzopyran-4-one (**2**) (peak 3, t_R_ = 13.9 min) were identified (Figure 1A and Figure 2). Besides these flavonoids (**1** and **2**), rutin or 2-(3,4-dihydroxyphenyl)-5,7-dihydroxy-3-[α-l-rhamnopyranosyl-(1→6)-β-d-glucopyranosyloxy]-4*H*-chromen-one (**3**) (peak 2, t_R_ = 7.8 min) was identified in EAEVP (Figure 1B and Figure 2).

### 2.2. Effect of HEVP on Different Irritant Agents-Induced Mice Ear Edema

**Figure 3:**
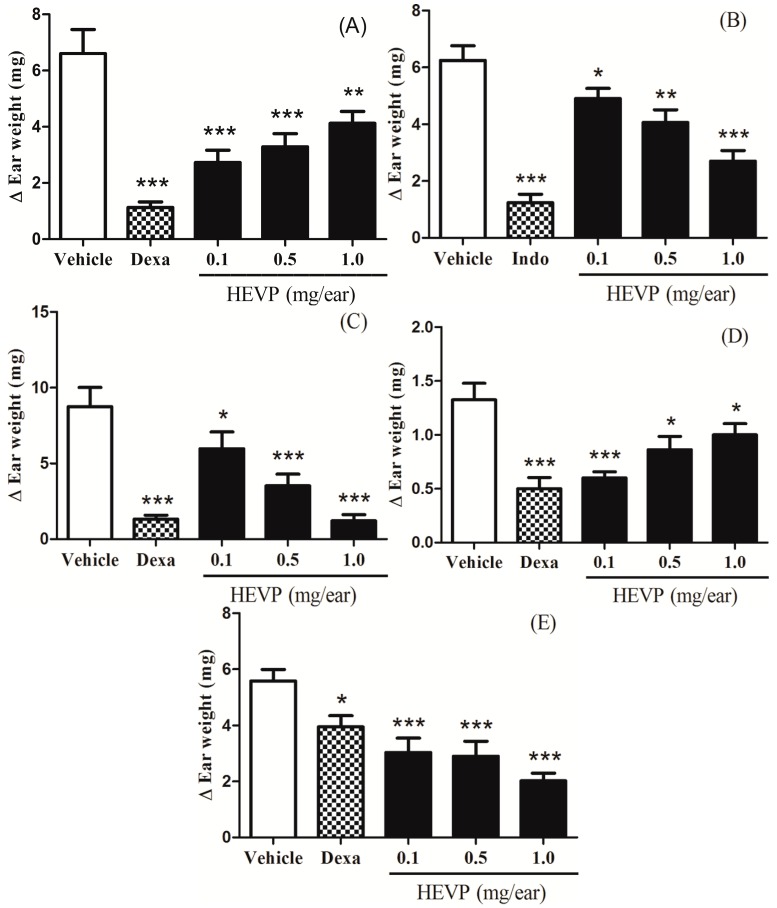
Topical effect of HEVP on mice ear edema induced by five different irritant agents. (**A**) Croton oil; (**B**) AA; (**C**) phenol; (**D**) EPP; and (**E**) capsaicin. Bars represent the mean ± S.E.M (*n* = 8). * *p* < 0.05, ** *p* < 0.01 and *** *p* < 0.001 represent the significance level when compared with negative control group. ANOVA followed by Student-Newman-Keuls test as post hoc.

The topical application of HEVP (0.1, 0.5 and 1.0 mg/ear) inhibited the ear edema induced by five different irritant agents when compared to negative control group (Figure 3). At the doses of 0.1, 0.5 and 1.0 mg/ear, HEVP reduced the ear edema induced by Croton oil in 58% (*p* < 0.001), 48% (*p* < 0.001) and 37% (*p* < 0.01), respectively (Figure 3A). The ear edema induced by arachidonic acid (AA) was inhibited in 21% (*p* < 0.05), 35% (*p* < 0.01), and 56% (*p* < 0.001) at the doses of 0.1, 0.5, and 1.0 mg/ear of HEVP, in this order (Figure 3B). After the treatment with 0.1, 0.5, and 1.0 mg/ear of HEVP, the ear edema induced by phenol was decreased in 31% (*p* < 0.05), 59% (*p* < 0.001), and 85% (*p* < 0.001), respectively (Figure 3C). The ear edema inhibition induced by ethyl phenylpropiolate (EPP) was 54% (*p* < 0.001), 34% (*p* < 0.05), and 23% (*p* < 0.05) at the doses of 0.1, 0.5, and 1.0 mg/ear of HEVP, in this order (Figure 3D). In addition, these results seem to indicate that the anti-inflammatory effect of HEVP is inversely dose-dependent for Croton oil and EPP (Figure 3A,D). After application of 0.1, 0.5, and 1.0 mg/ear of HEVP, the capsaicin-induced mice ear edema was reduced by 45% (*p* < 0.001), 47% (*p* < 0.001), and 63% (*p* < 0.001), respectively (Figure 3E). The anti-inflammatory drugs used as positive controls in these assays, dexamethasone 0.1 mg/ear (Croton oil, phenol, EPP, and capsaicin) or indomethacin 2.0 mg/ear (AA) were both effective (Figure 3).

### 2.3. Effect of EAEVP on Different Irritant Agents-Induced Mice Ear Edema

**Figure 4:**
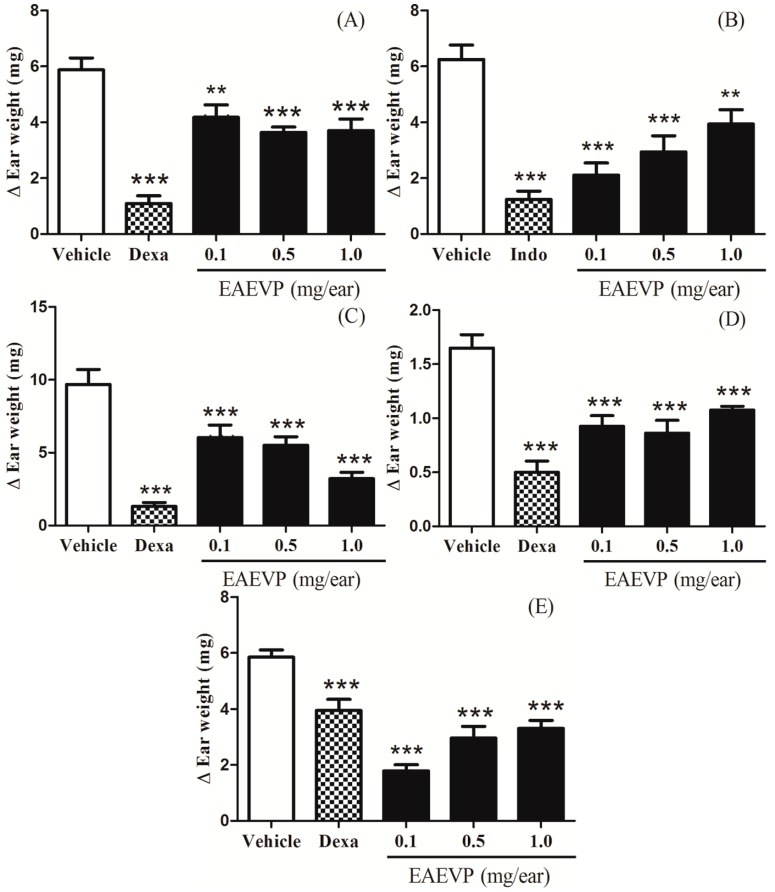
Topical effect of EAEVP on mice ear edema induced by five different irritant agents. (**A**) Croton oil; (**B**) AA; (**C**) phenol; (**D**) EPP; and (**E**) capsaicin. Bars represent the mean ± S.E.M (*n* = 8). ** *p* < 0.01 and *** *p* < 0.001 represent the significance level when compared with negative control group. ANOVA followed by Student-Newman-Keuls test as post hoc.

EAEVP (0.1, 0.5 and 1.0 mg/ear), as shown in Figure 4, when applied topically and compared to negative control group, significantly inhibited the edema caused by Croton oil (28%, *p* < 0.01; 37%, *p* < 0.001; 37%, *p* < 0.001, respectively, Figure 4A), AA (66%, *p* < 0.001; 52%, *p* < 0.001; 36%, *p* < 0.01, respectively, Figure 4B), phenol (37%, *p* < 0.001; 43%, *p* < 0.001; 66%, *p* < 0.001, respectively, Figure 4C), EPP (43%, *p* < 0.001; 47%, *p* < 0.001; 34%, *p* < 0.001, respectively, Figure 4D), and capsaicin (69%, *p* < 0.001; 49%, *p* < 0.001; 43%, *p* < 0.001, respectively, Figure 4E). In Figure 4B, the anti-inflammatory effect of EAEVP is also inversely dose-dependent for AA. The positive controls used in these assays, dexamethasone 0.1 mg/ear (Croton oil, phenol, EPP and capsaicin) or indomethacin 2.0 mg/ear (AA), were significantly active against ear edema (Figure 4).

### 2.4. Histopathology Analysis

**Figure 5:**
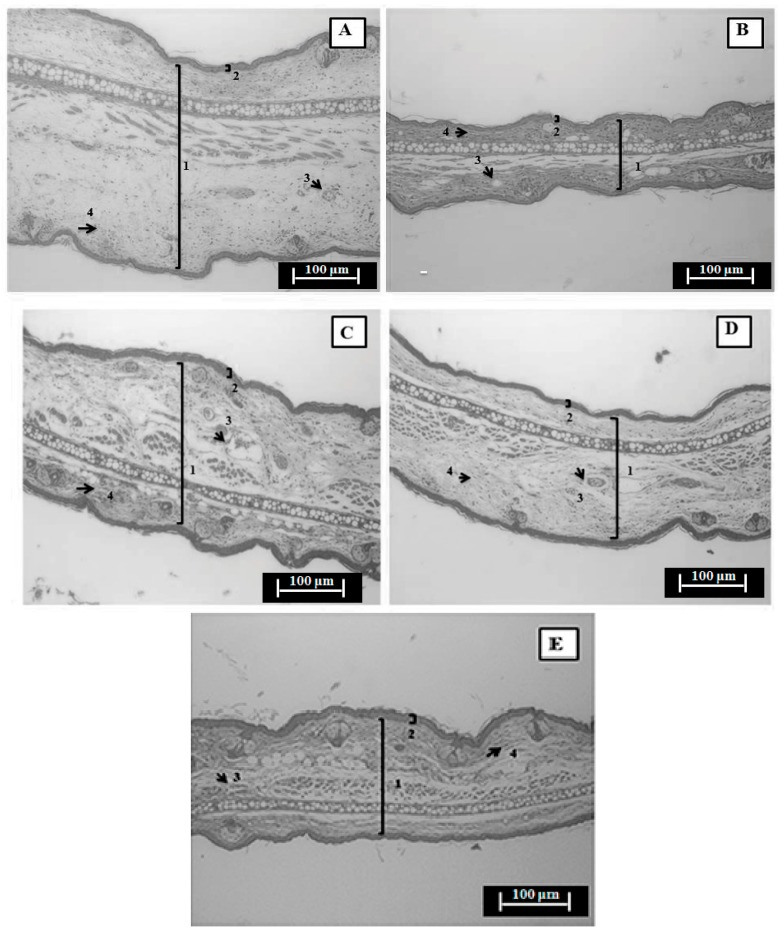
Photomicrograph of transverse sections of H&E-stained mice ear tissue sensitized with topical single application of Croton oil examined under qualitative light microscopy (magnification: 200×, scale: 100 µm). (**A**) Negative control group (Croton oil in 20 µL acetone); (**B**) Non-inflamed ear (acetone); (**C**) HEVP (0.1 mg/ear), (**D**) EAEVP (0.5 mg/ear); and (**E**) Positive control group (dexamethasone, 0.1 mg/ear). Numbers 1 and 2 indicate the ear thickness (dermis increase) and epidermal, respectively. Arrows numbered 3 and 4 indicate inflammatory cells (infiltration of mononuclear leukocytes) and blood vessel (vasodilation), respectively.

The histopathological evaluation of the ears submitted to a single Croton oil application revealed a significant increase in the dermis thickness with edema formation (untreated group), which was accompanied by vascular congestion/vasodilation and infiltration of inflammatory cells (Figure 5A). In addition, the physiological characteristics were preserved in the normal tissue (non-inflamed) (Figure 5B). When compared to the negative control group, HEVP 0.1 mg/ear (Figure 5C), EAEVP 0.5 mg/ear (Figure 5D) and dexamethasone 0.1 mg/ear (Figure 5E) decreased the ear thickness, inflammatory cells infiltration and vasodilation (Figure 5A). The ear thickness measurements (mm) showed that HEVP, EAEVP, and dexamethasone also reduced the edematous tissue thickness, confirming the anti-inflammatory effect (Table 1).

### 2.5. Effect of HEVP and EAEVP on MPO Activity in Croton Oil-Induced Ear Edema

**Figure 6:**
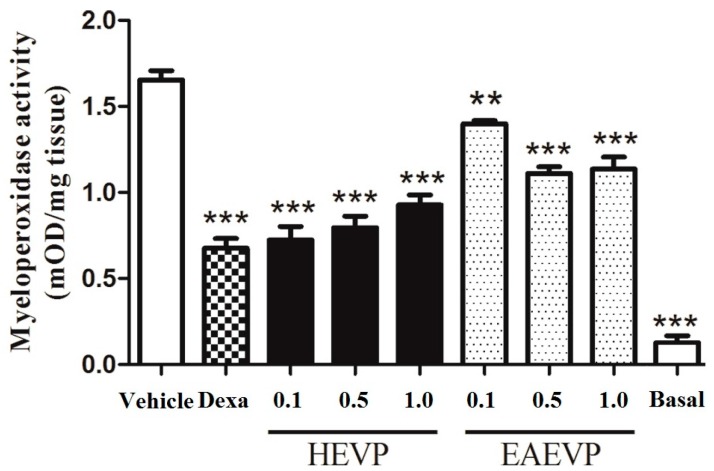
Effect of HEVP, EAEVP, and dexamethasone (Dexa) on MPO activity in Croton oil-induced ear edema. Bars represent the mean ± S.E.M. of 5 animals. ** *p* < 0.01 and *** *p* < 0.001 represent the significance level when compared with control group. ANOVA followed by Student-Newman-Keuls test as post hoc.

The topical application of Croton oil intensively increased the MPO activity, which was significantly inhibited by HEVP, EAEVP, and dexamethasone (0.1 mg/ear) with basal value equal to 0.13 ± 0.04 mOD/mg tissue (Figure 6). At the doses of 0.1, 0.5, and 1.0 mg/ear, HEVP inhibited the MPO activity by 56, 51, and 43% (*p* < 0.001), respectively. After treatment, EAEVP was able to reduce the MPO activity with 15 (*p* < 0.01), 33 (*p* < 0.001), and 31% (*p* < 0.001) of inhibition at the doses of 0.1, 0.5, and 1.0 mg/ear, respectively. Dexamethasone 0.1 mg/ear inhibited the MPO by 58%.

### 2.6. Effect of HEVP and EAEVP on NAG Activity in Croton Oil-Induced Ear Edema

**Figure 7:**
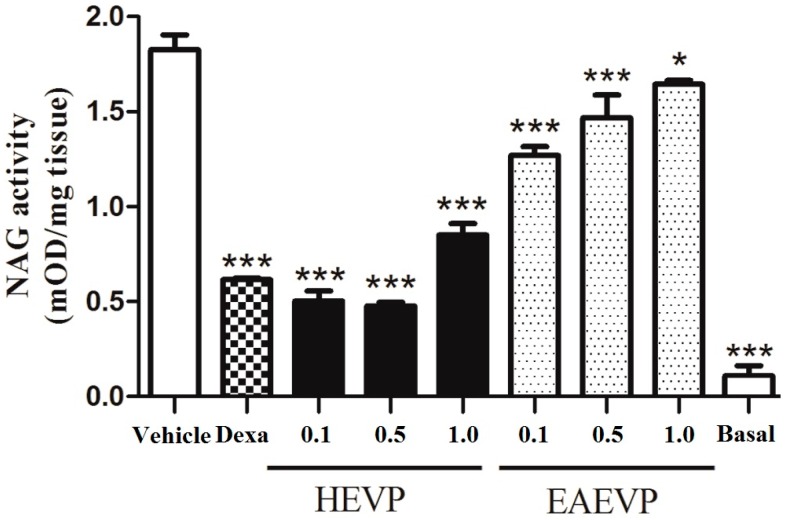
Effect of HEVP, EAEVP, and dexamethasone (Dexa) on NAG activity in Croton oil-induced ear edema. Bars represent the mean ± S.E.M. of 5 animals. * *p* < 0.05 and *** *p* < 0.001 represent the significance level when compared with control group. ANOVA followed by Student-Newman-Keuls test as post hoc.

The NAG activity was also increased after application of Croton oil, which was significantly inhibited by the extracts and dexamethasone (0.1 mg/ear) and presented basal value of 0.11 ± 0.05 mOD/mg tissue (Figure 7). When treated with HEVP, NAG activity was inhibited by 71.7%, 73.5%, and 53.0% (*p* < 0.001) at the doses of 0.1, 0.5, and 1.0 mg/ear, respectively. NAG activity was also inhibited at doses of 0.1 (30%; *p* < 0.001), 0.5 mg/ear (20%; *p* < 0.001), and 1.0 mg/ear (10%; *p* < 0.05) after treatment with EAEVP. Dexamethasone was active against NAG with 66% of inhibition.

## 3. Discussion

Ear edema models induced by phlogistic agents have been extensively used as pharmacological tools for the investigation of new topical anti-inflammatory drugs, including natural products that are useful in the treatment of inflammatory skin disorders [27]. In this study, the authors showed for the first time that HEVP and EAEVP have topical anti-inflammatory effect, which is possibly related to the presence of secondary compounds, since some flavonoids (rutin, luteolin, and apigenin) were identified in EAEVP and this effect may involve different mechanisms in the cutaneous inflammation [28,29]. Rutin, for example, inhibited the xilol-induced ear edema and reduced the cell migration and the levels of cytokines were also observed [30], while luteolin exhibits anti-inflammatory effects by blocking the activity of heat shock protein 90 in macrophages [31]. In addition, apigenin has been effective against the LPS-induced acute lung injury due to its ability of primary inhibition of cyclooxygenase-2 (COX-2) gene expression and nuclear factor kB (NF-kB) gene expression and the protective mechanism of apigenin may be attributed partly to decreased production of proinflammatory cytokines [32].

Firstly, the authors studied the topical anti-inflammatory effect of HEVP and EAEVP in Croton oil-induced mice ear edema. Croton oil comprises a mixture of lipids, which has 12-*O*-tetracanoilphorbol-13-acetate (TPA), an ester of phorbol, as main component, that activate the phospholipid-dependent protein kinase C (PKC) to stimulate mitogen activated protein kinases (MAPK), and phospholipase A2 (PLA2) with the release of platelet activation factor (PAF) and AA [33]. Such enzymes and metabolic products generate mediators that promote vascular permeability, vasodilation, leukocyte migration, release of histamine and serotonin (5-HT), and synthesis of prostaglandins (PG) and leukotrienes (LT). In addition, the activation of nuclear transcription factors (e.g., NF-κB and AP-1) by MAPK regulates the inflammatory process through their direct stimulation of pro-inflammatory cytokines, TNFα, interleukins (ILs), and metalloproteinases [34]. Considering these aspects, non-steroidal anti-inflammatory drugs (NSAIDs), corticosteroids, and leukotriene receptor antagonists have been effective against the inflammation induced by Croton oil and TPA [35,36]. As first evidence, our results showed that HEVP and EAEVP were active against Croton oil-induced ear edema by reducing the inflammatory parameters (Figure 3A, Figure 4A and Figure 5, and Table 1) triggered by this agent. When administered topically, HEPV significantly reduced the ear edema (*p* < 0.001 or *p* < 0.01) regardless of the doses (0.1; 0.5 or 1.0 mg/ear), as well as the dexamethasone was more efficient than either dose (*p* < 0.001). However, the anti-inflammatory action of this extract was not dose-dependent (Figure 3A) suggesting a possible antagonism between the components of the extract associated with the increase of the dose [37,38,39].

AA has been implicated in the pathogenesis of skin diseases and its topical application generates inflammatory eicosanoids (prostaglandin E2 and leukotrienes) and histamine with formation of erythema, edema, blood flow, vascular permeability, and neutrophil accumulation [40,41,42]. In the current study, HEVP and EAEVP were effective in decreasing the AA-induced ear edema producing an anti-edematous effect (Figure 3B and Figure 4B) as observed with the positive control (indomethacin), which is a cyclooxygenase inhibitor. In addition, even with significant (*p* < 0.001 or *p* < 0.01) reduction in the doses tested, the inhibition of the ear edema by EAEVP was not dose-dependent (Figure 4B). As described for the Croton oil, this phenomenon may be related to antagonism between the substances of the extract, which is evidenced with increasing dose [37,38,39].

Phenol produces an inflammatory response with characteristic of contact dermatitis to stimulate the breakdown of keratinocytes with release of cytokines (IL-1α, TNF-α and IL-8) [43,44]. Moreover, other mediators such as PG and reactive oxygen species (ROS) are also involved in this inflammatory response [35]. Our results showed that HEVP and EAEVP significantly reduced the phenol-induced edema (Figure 3C and Figure 4C) in a dose-dependent manner similarly to dexamethasone. The antioxidant capacity of flavonoids supported by the presence of apigenin, luteolin, and rutin via HPLC in these extracts may also potentiate this effect. Therefore, this finding can indicate the potential use of HEVP and EAEVP for the treatment of contact dermatitis.

EPP is an irritant that can promote ear edema by release of histamine, serotonin (5-HT), bradykinin (BK), and PG, followed by vasodilation and increase of vascular permeability [45,46]. It was found that both extracts (HEVP and EAEVP), as well as dexamethasone, elicited a significant inhibitory effect on the edema formation (Figure 3D and Figure 4D). In this sense, this effect can be due to inhibition of the release or synthesis of inflammatory mediators involved in EPP action. Dexamethasone was very effective in this model, but the inhibition of the ear edema was not completely dependent on dose of HEVP and EAEVP. The antagonism between the constituents of the extract can also justify this finding, as mentioned above [37,38,39].

Capsaicin is a capsaicinoid that causes a cutaneous neurogenic inflammation after activation of the vanilloid receptor subtype 1 (TRPV1) with generation of substance P (SP) and calcitonin gene related peptide (CGRP) and release of proinflammatory mediators, such as BK, histamine, 5-HT, and PG [47,48]. HEVP and EAEVP markedly inhibited the capsaicin-induced ear edema probably through the blockade of TRPV1 receptors by preventing the spread of painful stimuli. This data supported the previous report of the antinociceptive activity of *V. polyanthes* made by our research group [17]. Although dexamethasone is commonly used in this assay, it is not very effective in reducing the edema induced by capsaicin.

The results observed in the histopathological analysis of ear tissue sections obtained from Croton oil-induced edema corroborated by HEVP and EAEVP antiedematous effect (Figure 3A and Figure 4A), since they demonstrated the reduction of dermis thickness (edema), vasodilation, and leukocytes infiltration (Figure 5 and Table 1).

For better understanding of the topical anti-inflammatory effect, HEVP and EAEVP were tested for MPO and NAG activities of the Croton oil-induced mice ear edema. In the inflammatory processes, MPO is an enzyme localized in the intracellular granules of neutrophils and plays a major role in the metabolic activity of these cells [49]. The authors determined MPO activity as an indirect measure of neutrophil migration into the interstitial space and inflamed tissue content, since the enzymatic activity correlates with the number and activation state of polymorphonuclear cells. Our results showed a high reduction of MPO activity by HEVP and EAEVP that can be associated with the anti-inflammatory action of these extracts. In addition, these data become relevant since MPO has been implicated in several pathologies, including atherosclerosis, myocardial infarction, atrial fibrillation, multiple sclerosis, Alzheimer’s disease, lung cancer, and transplant rejection [49,50,51].

NAG is a lysosomal enzyme produced by activated macrophages and the measure of its activity is considered an indirect indicator of the presence of mononuclear cells in the inflammatory site. Although these cells may be found in the acute inflammatory process, it is important to mention that its elevation is a characteristic of chronic inflammation [52]. Considering this context, our results showed that HEVP and EAEVP decreased NAG activity indicating a reduction of the migration of mononuclear cells and alleviation of symptoms caused by inflammatory cells. Therefore, the inhibition of NAG activity confirmed the topical anti-inflammatory effect described above.

From the chemical viewpoint, flavonoids, a relevant group of natural compounds, identified by HPLC-UV/DAD, have been related to anti-inflammatory and antioxidant mechanisms [24,25]. Anti-inflammatory mechanisms of these compounds, including rutin and luteolin, are associated with inhibition of enzymes such as PLA2, cyclooxygenases, and lipoxygenases reducing the generation of prostanoids and leukotrienes [53,54,55]. In addition, flavonoids inhibit histamine release, phosphodiesterase, protein kinases, and activation of transcriptase [54,55]. Rutin is able to suppress the production of tumor necrosis factor-α and interleukin 6 and the activation of nuclear factor-κB and extracellular regulated kinases 1/2, which may explain the anti-inflammatory effect writing [56]. Thus, the phytochemicals of *V. polyanthes* are probably interacting with important inflammatory pathways preventing the actions of mediators implicated in the formation of edema, and can be potential targets for the development of new topical anti-inflamatory therapeutic agents.

## 4. Materials and Methods

### 4.1. Plant Material

*V. polyanthes* was cultivated and collected in the Medicinal Garden of the Faculty of Pharmacy, Federal University of Juiz de Fora, Juiz de Fora city, Minas Gerais State, Southeast region of Brazil, in March 2012. A voucher specimen, identified by Fátima Regina Gonçalves Salimena, was deposited in the Herbarium of the Federal University of Juiz de Fora (CESJ number 10.329). The leaves were placed in a drying oven with forced air circulation at 50 °C for a loss of 90%–95% humidity.

### 4.2. Chemicals

Drugs and reagents used in this study (and their sources) were as follows: Croton oil (≈100%), arachidonic acid (AA, ≥99%), phenol (≥99%), capsaicin (≥99%), ethyl phenylpropiolate (EPP, 98%), dexamethasone (Dexa, ≥97%), indomethacin (Indo, ≥99%), phosphoric acid (85%), rutin hydrate (≥94%), quercetin (≥95%), kaempferol (≥99%), apigenin (≥99%), apigenin7-*O*-β-d-glucoside (≥98%), luteolin (≥97%), luteolin 7-*O*-β-d-glucoside (≥98%), hexadecyltrimethylammonium bromide (≥99%), 3,3′,5,5′-tetramethylbenzidine dihydrochloride hydrate (≥98%), *p*-nitrophenyl-acetamide-µ-d-glucopyranoside (≥98%), sodium phosphate (96%), glycine (≥98.5%), and sodium citrate dehydrate (≥99%) (Sigma-Aldrich Co., St. Louis, MO, USA), dimethyl sulfoxide (≥99.7%), hexane (≥99%), ethyl acetate (≥99%), and acetone (≥99%) (Vetec Química Farm Ltda, Rio de Janeiro, RJ, Brazil), acetonitrile (≥99.9%) (Tedia, Brazil), and ketamine chloride (10%) and xylazine chloride (2%) (Syntec, Hortolândia, SP, Brazil).

### 4.3. Extract Preparation

**Figure 8:**
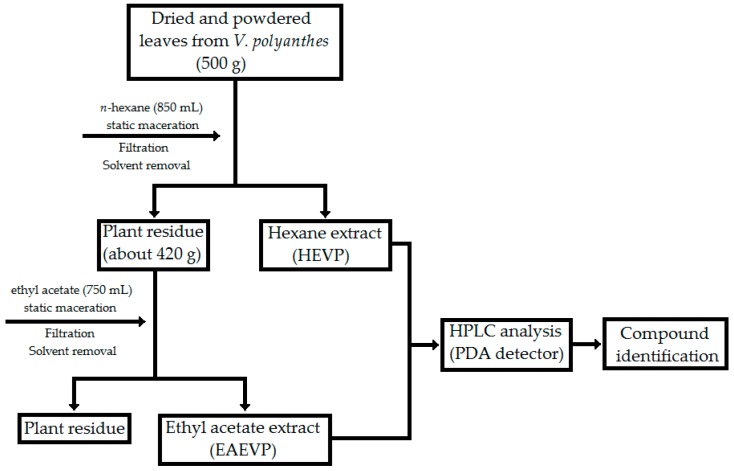
Flowchart of the extraction process from *V. polyanthes* leaves.

Initially, dried and powdered leaves (500 g) were exhaustively extracted with 850 mL of *n*-hexane by static maceration for three months at room temperature with renewal of solvent every three days. After filtration, the liquid hexane extract was evaporated under a rotary evaporator (R-215 Büchi Labortechnik AG, Flawil, Switzerland) at controlled temperature (40–50 °C), and the dry hexane extract (HEVP) was obtained. The material resulting (about 420 g) from this process was subjected to new extraction with 750 mL of ethyl acetate to acquire the ethyl acetate extract (EAEVP) using the same procedure for two months (Figure 8).

### 4.4. High-Pressure Liquid Chromatography (HPLC) Analysis

HPLC analysis was performed using an Agilent Technologies 1200 Series, with a PDA detector and an automatic injector. The column employed was a Zorbax SB-18; 250 × 4.6 mm, 5 μm particle sizes. The mobile phase used was water/acetonitrile (*v*/*v*) 0–10 min, 90:10; 10–12 min, 70:30; 12–32 min, 60:40. The elution flow was 1 mL/min and 20 µL of HEVP and EAEVP (1 mg/mL) was injected. Chromatograms were obtained using 190 to 400 nm wavelength range. Pure compounds, such as gallic acid, rutin, quercetin, kaempferol, luteolin, luteolin7-*O*-β-d-glucoside, apigenin, and apigenin7-*O*-β-d-glucoside, were used as chemical markers. The identification of the compounds was performed by comparison of the retention time (t_R_) and UV spectrum with co-injection of the chemical markers.

### 4.5. Pharmacological Assays

#### 4.5.1. Animals

In this study, male Swiss albino mice (*Mus musculus*) (45–55 days) weighing 25–30 g were used from the Central Biotery of the Center for Biology of Reproduction of the Federal University of Juiz de Fora, and the procedures were performed in the Laboratory of Pharmacology of Natural Products of this Institution. The animals were divided into groups and kept in plastic cages (47 × 34 × 18 cm) at room temperature (22 ± 2 °C), with free access to Purina^®^ chow and water. Animal care and the experimental protocol followed the principles and guidelines suggested by the Brazilian College of Animal Experimentation (COBEA) and were approved by the Institutional Ethics Committee (protocol number 106/2012, 05 March 2013).

#### 4.5.2. Croton Oil-Induced Ear Edema

Croton oil-induced ear edema in mice was performed as recommended by Schiantarelli et al. [57]. Ear edema was induced (*n* = 8/group) by topical application of 20 μL of Croton oil 2.5% (*v*/*v*) in acetone on the inner surface of the right ear, while the left side received 20 μL of acetone (vehicle). After 15 min, HEVP and EAEVP (0.1, 0.5 and 1.0 mg/ear) and dexamethasone (0.1 mg/ear, positive control), dissolved in 20 μL of acetone, were topically applied on the right ear. The negative control group received 20 μL of acetone on the left ear. The ear edema was evaluated 6 h after Croton oil application and was expressed as increase in ear weight (mg). Ear tissue samples (6 mm) were conserved in formaldehyde 10% (*v*/*v*) and submitted to histopathological analysis.

#### 4.5.3. AA-, Phenol-, and EPP-Induced Ear Edema

Edema was induced in mice (*n* = 8/group) by topical administration on the inner surface of the right ear using the following irritant agents: AA (2.0 mg/ear in 20 μL of acetone) [40], phenol (10% *v*/*v* in 20 μL of acetone) [58] and EPP (1.0 mg/ear in 20 μL of acetone) [59], while the left ear received 20 μL of acetone as vehicle. Fifteen min after induction of the edema, the right ear was topically treated with HEVP or EAEVP (0.1, 0.5 and 1.0 mg/ear in 20 μL of acetone), indomethacin (2.0 mg/ear in 20 μL of acetone, positive control for AA), and dexamethasone (0.1 mg/ear 20 μL of acetone, positive control for phenol and EPP). Acetone (20 μL/ear, negative control) was topically applied on the left ear. The ear edema was evaluated 1 h after AA and EPP, and 2 h after phenol application by means of increase in ear weight (mg).

#### 4.5.4. Capsaicin-Induced Ear Edema

Capsaicin-induced ear edema in mice (*n* = 8/group) was performed according Gábor and Razga’s procedure [60]. Thirty min after the topical treatment of HEVP and EAEVP (0.1, 0.5, and 1.0 mg/ear in 20 μL of acetone), dexamethasone (0.1 mg/ear 20 μL of acetone, positive control), and acetone (20 μL/ear, negative control), capsaicin (200 μg/ear in 20 μL of acetone) was topically applied on the inner surface of the right ear. The left ear received 20 μL of acetone as vehicle. The ear edema was evaluated after 30 min of capsaicin application based on the increase of ear weight (mg).

#### 4.5.5. Ear Edema Evaluation

After induction and treatment of the edema, the animals of each model were euthanized with a solution of ketamine (240 mg/kg) and xylazine (45 mg/kg) and 6 mm diameter ear tissue samples were collected from right and left ears using a metallic punch (Richter). Then, the tissue samples were individually weighed on analytical balance (AY220, Shimadzu) and the edema was evaluated by the difference of weight (mg) between right and left (non-inflamed) ears.

#### 4.5.6. Histopathological Analysis

As described by Chibli et al. [35], ear tissue samples (circles of 6 mm) obtained from Croton oil-induced ear edema model were preserved in formaldehyde 10% (*v*/*v*) and fixed in 70% ethanol for 24 h. These samples were dehydrated, blocked in paraffin, and then sectioned with a microtome (5 μm) (TBS Cut 4060 Rotary Microtome, Thermo Fisher Scientific Inc., Pittsburgh, PA, USA). The transverse sections were stained with hematoxylin and eosin (H&E) for the evaluation of edema (dermis) thickness, epidermal hyperplasia, inflammatory cell infiltration, and vasodilation. Representative areas were selected for qualitative light microscopic analysis (BX 51 Olympus microscopy, Olympus Optical Co., Ltd., Tokyo, Japan; magnification: 200×) and images were captured (photomicrograph) through the Image-Pro^®^ Plus software (version 6.0, Media Cybernetics Inc., Rockville, MD, USA), which was also used to acquire measurements of ear thickness (μm) of tissue samples. A representative section from each group of animals was selected to show the histopathological changes.

#### 4.5.7. Tissue Myeloperoxidase (MPO) Assay

MPO activity was evaluated from Croton oil-induced ear edema samples [61]. The samples (*n* = 5) were placed into 0.75 mL of 80 mM sodium phosphate buffer (PBS, pH 5.4) containing 0.5% hexadecyltrimethylamonium bromide (HTAB) and homogenized (45 s at 0 °C) in a motor-driver homogenizer. The homogenate was decanted into a microcentrifuge tube, and the vessel was washed with a second 0.75 mL aliquot of HTAB in PBS that was added to the tube. This mixture (1.5 mL) was centrifuged at 12,000× *g* at 4 °C for 15 min. After centrifugation, the supernatant samples (triplicates of 25 µL) were added to 96-well microliter plates containing 25 µL of 1.6 mM tetramethylbenzidine HCL (TMB) in DMSO and 100 µL of 0.003% of H_2_O_2_ were added to the wells. The plates were incubated for 5 min at 37 °C, and the reaction was subsequently stopped with addition of 100 µL 4.0 M sulfuric acid at 4 °C. Enzyme activity was determined colorimetrically using a plate reader (EL808B, Bio Tek Instruments Inc., Winooski, VT, USA) to measure absorbance at 450 nm, and the results were expressed as mOD/mg tissue.

#### 4.5.8. Tissue *N*-Acetyl-β-d-glucosaminidase (NAG) Assay

According to the method used by Lloret and Moreno [62], ear samples (circles of 6 mm) were treated using the same method described for the MPO assay. The supernatant samples (triplicates of 100 µL) were added into 96-well microtiter plates. For the assay, 100 µL of *p*-nitrophenyl-acetamide-µ-d-glucopyranoside (2.24 mM) dissolved in 0.1 M buffer citrate (pH 4.5) per well were used. The mixture was incubated for 60 min at 37 °C, and the reaction was stopped by the addition of 100 µL of 0.2 M buffer glycine (pH 10.6). The enzyme activity was determined colorimetrically using a plate reader (EL808B, Bio Tek Instruments Inc., Winooski, VT, USA) to measure absorbance at 405 nm, and enzyme activity was expressed as mOD/mg tissue.

### 4.6. Statistical Analysis

Data were presented as mean ± S.E.M. Analysis of variance (ANOVA) followed by post hoc Student-Newman-Keuls test was used to determine the significant level (*p* < 0.05) using the Graph Pad Prism^®^ Software (version 5.0, Graph Pad Software Inc., La Jolla, CA, USA).

## 5. Conclusions

In conclusion, the current study showed that HEVP and EAEVP have a relevant topical anti-inflammatory effect using ear edema models, which can be related to inhibition of several inflammatory mediators by secondary metabolites as demonstrated in the tests performed. In addition, these findings corroborate with the popular use of the species for the treatment of inflammation and cutaneous damage, and also contribute to the international policy strategies proposed by World Health Organization related to Traditional and Complementary Medicine (T&CM).

## Figures and Tables

**Table 1:** Effect of HEVP and EAEVP on ear edema thickness induced by Croton oil.

Group	Dose	Ear Thickness (µm)	Edema Inhibition (%)
Non-inflamed	-	192.0 ± 7.0	-
Acetone (vehicle)	20 µL/ear	705.3 ± 11.5	0
	0.1 mg/ear	389.7 ± 6.7 ***	58.8%
HEVP	0.5 mg/ear	429.5 ± 3.2 ***	50.3%
	1.0 mg/ear	472.6 ± 9.8 ***	37.6%
	0.1 mg/ear	540.1 ± 29.1 ***	19.9%
EAEVP	0.5 mg/ear	528.7 ± 32.5 ***	22.8%
	1.0 mg/ear	543.1 ± 24.3 ***	22.3%
Dexamethasone	0.1 mg/ear	236.6 ± 8.9 ***	79.5%

Data expressed as mean ± S.E.M. Measurements were made at three different points of each section and each group was represented by four sections (*n* = 4). *** *p* < 0.001 represents the significance level when compared with the negative control group. ANOVA followed by Student-Newman-Keuls test as post hoc.

## Abbreviations

HEVP	Hexane extract from *Vernonia polyanthes* leaves
EAEVP	Ethyl acetate extract of *Vernonia polyanthes* leaves
HPLC–UV–DAD	high-performance liquid chromatography–ultraviolet–diode-array detection
AA	Arachidonic acid
EPP	Ethyl phenylpropiolate
MPO	Myeloperoxidase
NAG	*N*-acetyl-β-d-glucosaminidase
